# Characterization of Genes That Exhibit Genotype-Dependent Allele-Specific Expression and Its Implications for the Development of Maize Kernel

**DOI:** 10.3390/ijms24054766

**Published:** 2023-03-01

**Authors:** Xiaomei Dong, Haishan Luo, Jiabin Yao, Qingfeng Guo, Shuai Yu, Xiaoyu Zhang, Xipeng Cheng, Dexuan Meng

**Affiliations:** 1 College of Bioscience and Biotechnology, Shenyang Agricultural University, Shenyang 110866, China; 2 Shenyang City Key Laboratory of Maize Genomic Selection Breeding, Shenyang 110866, China; 3 College of Agronomy, Shenyang Agricultural University, Shenyang 110866, China

**Keywords:** maize, heterosis, kernel development, allelic expression, epigenetic regulation

## Abstract

Heterosis or hybrid vigor refers to the superior phenotypic traits of hybrids relative to their parental inbred lines. An imbalance between the expression levels of two parental alleles in the F1 hybrid has been suggested as a mechanism of heterosis. Here, based on genome-wide allele-specific expression analysis using RNA sequencing technology, 1689 genes exhibiting genotype-dependent allele-specific expression (genotype-dependent ASEGs) were identified in the embryos, and 1390 genotype-dependent ASEGs in the endosperm, of three maize F1 hybrids. Of these ASEGs, most were consistent in different tissues from one hybrid cross, but nearly 50% showed allele-specific expression from some genotypes but not others. These genotype-dependent ASEGs were mostly enriched in metabolic pathways of substances and energy, including the tricarboxylic acid cycle, aerobic respiration, and energy derivation by oxidation of organic compounds and ADP binding. Mutation and overexpression of one ASEG affected kernel size, which indicates that these genotype-dependent ASEGs may make important contributions to kernel development. Finally, the allele-specific methylation pattern on genotype-dependent ASEGs indicated that DNA methylation plays a potential role in the regulation of allelic expression for some ASEGs. In this study, a detailed analysis of genotype-dependent ASEGs in the embryo and endosperm of three different maize F1 hybrids will provide an index of genes for future research on the genetic and molecular mechanism of heterosis.

## 1. Introduction

Heterosis or hybrid vigor refers to the superior phenotypic traits of hybrids relative to their parental inbred lines [1]. Phenotypic traits include plant height, development rate, male and female fertility, nutrient quality, grain yield, and tolerance to stress. This phenomenon was first described by Charles Darwin and was later independently rediscovered by George H. Shull and Edward M. East in 1908. In the last few hundred years, heterosis has been widely exploited to increase crop yield and improve agricultural production [2,3,4,5,6].

Although not well understood at the molecular level, heterosis has been exploited over the past half-century in plants and animals [7,8]. Extensive studies on heterosis using RNA sequencing (RNA-seq) technologies have identified differentially expressed genes (DEGs) between F1 hybrids and their parental inbred lines in plants [9,10,11,12,13,14,15,16]. For example, in maize reciprocal F1 hybrids, a total of 1510 and 647 genes showed additive expression in shoots and roots, respectively [17]. Allele-specific expression (ASE) refers to the specific or preferential expression of one parental allele in the hybrid due to variations in regulatory sequences between the maternal and paternal genomes. The detection of single nucleotide polymorphisms (SNPs) in parent lines can be used to distinguish parental alleles and identify genes showing ASE in heterozygotes. To date, ASE has also been analyzed in several plants, including Arabidopsis, rice, maize, and barley [17,18,19,20,21]. For example, in rice, 23.8% of genes showed a preferential allele expression that was genotype-dependent in leaves of reciprocal crosses [16]. ASE accounted for 79.8% of the genes that showed more than a 10-fold expression level difference between an F1 and its parents. The expression difference caused by ASE may lead to phenotypic variation depending on the function of the genes. Several studies have suggested that ASE plays a role in heterosis because genetic variations often cause differences in gene expression, which may lead to phenotypic variations [22]. Genes showing allele-specific expression can lead to heterosis-relevant phenotype variation [23].

In addition to genetic variations, epigenetic variations have been suggested to play a role in the regulation of differential gene expression in plant hybrids, leading to the hybrid phenotype [24,25,26,27]. Genome activity and chromatin states can be regulated by epigenetic modifications in eukaryotes, mainly DNA methylation and histone modifications [28]. DNA cytosine methylation, as an important epigenetic modification, occurs in the context of CG, CHG, and CHH (where H is A, C, or T) in plants. The major role of DNA cytosine methylation is to silence transposable elements (TE) and repetitive sequences and suppress gene promoter activity [29,30,31]. Genome-wide allele-specific DNA methylation has been investigated in plants, including Arabidopsis, rice, and maize [32,33,34,35]. In endosperm, differential levels of DNA methylation have been observed around imprinted genes and are essential for allele-specific expression of imprinted genes [36,37]. Recently, in rice, DNA methylation differences between two inbred lines, ZS97 and MH63, and parental methylation interactions in reciprocal hybrids were investigated [38]. The results revealed a specific role for the divergence of parental CHG methylation in ASEGs, which is associated with phenotype variation and hybrid vigor in several plant species. Maize is an ideal model system for the study of ASEGs in hybrids due to its significant heterotic performance and well-known complex genome [39,40]. In this study, using RNA sequencing technology, we systematically identified genes exhibiting genotype-dependent ASEGs in embryos and endosperm from three maize F1 hybrids. Comparison of the allelic expression of these ASEGs in different tissues and hybrid crosses suggests that these ASEG patterns may have distinct implications for the genetic and molecular basis of heterosis. Further functional analysis indicated that these genotype-dependent ASEGs may make important contributions to kernel development. Finally, the potential relationship between DNA methylation and genotype-dependent ASEGs was also investigated.

## 2. Results

### 2.1. Identification of ASEGs in the Embryo and Endosperm of Three Reciprocal Crosses

To explore global ASEGs in hybrid maize and reveal the mechanism of differential expression in the embryo and endosperm of F1 hybrids, three maize inbred lines (B73, Mo17, and CAU5) were chosen to generate three reciprocal crosses, B73 × Mo17 (BM) and Mo17 × B73 (MB), B73 × CAU5 (BC) and CAU5 × B73 (CB), and Mo17 × CAU5 (MC) and CAU5 × Mo17 (CM). RNA sequencing (RNA-seq) of the immature embryo and endosperm at 11 days after pollination (DAP) of three reciprocal crosses was performed.

**Figure 1:**
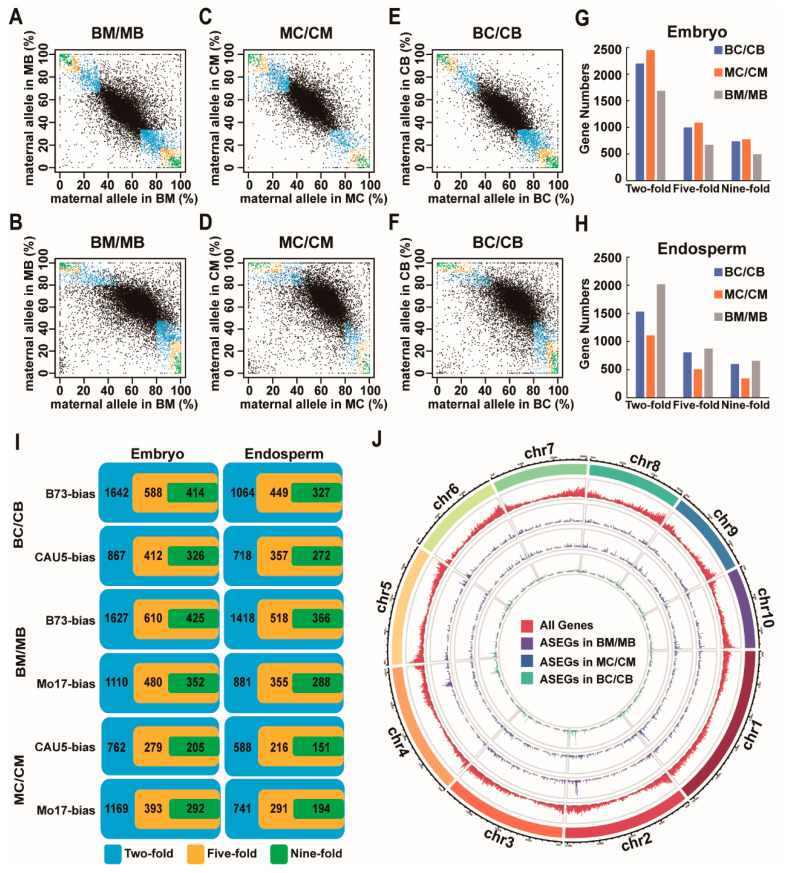
Identification and genome distribution of genotype-dependent ASEGs. (**A**–**F**) Maternal reads of the proportion of genes in embryo and endosperm from three reciprocal hybrids. The black dots represent all the analyzed genes except for ASEGs. The blue, orange, and green dots represent the ASEGs identified under two-fold, five-fold, or nine-fold criteria. (**G**,**H**) The number of ASEGs identified in the embryo (**G**) and endosperm (**H**). (**I**) The number of genes that preferred to express the B73 allele, the CAU5 allele, or the Mo17 allele in embryo and endosperm from three reciprocal hybrids. (**J**) Chromosomal distribution of ASEGs identified in three reciprocal crosses. The red, purple, blue, and green lines represent, respectively, all genes, and ASEGs identified in BM/MB, MC/CM, and BC/CB.

Here, a combination of proportion filters and statistical significance was applied to identify and classify genes that exhibit genotype-dependent allele-specific expression in the study (see Section 4). As illustrated in Figure 1A–F, most genes exhibited the expected maternal-to-paternal ratio of 1:1 or 2:1 in the embryo or endosperm (q > 0.05, x^2^ test). Based on statistically significant deviation (q < 0.05, x^2^ test), read counts from one parental allele being at least two-fold, five-fold, or nine-fold higher than read counts from another parental allele were used to identify ASEGs (Figure 1G–I). Under the criteria of a nine-fold difference between reads from two parents, a total of 740, 497, and 777 genes showed ASE in the embryos from BC/CB, MC/CM, and BM/MB, respectively (Figure 1G; Tables S1–S3). A total of 599, 347, and 657 genes showed ASE in endosperm from BC/CB, MC/CM, and BM/MB, respectively (Figure 1H; Tables S1–S3). These ASEGs were further classified according to which parent they preferred to express (Figure 1I). For example, according to criteria with a nine-fold difference, 740 ASEGs in the BC/CB embryos included 414 genes that preferred to express the CAU5 allele and 326 genes that preferred to express the B73 allele, and 599 ASEGs in endosperm from BC/CB included 327 genes that preferred to express the CAU5 allele and 272 genes that preferred to express the B73 allele (Figure 1I).

In the BM/MB hybrid cross, the number of ASEGs was the largest in both the embryo and the endosperm (Figure 1G,H). The differences in the number of ASEGs for the three hybrid crosses were largely due to differences in the number of genes with polymorphisms. Using the circus program, the chromosomal locations of the ASEGs were detected in three hybrid crosses, and these ASEGs were evenly distributed in all chromosomes without obvious location preference (Figure 1J). The average distance between ASEGs was 2.23 Mb in BC/CB, 3.33 Mb in MC/CM, and 2.23 Mb in BM/MB. Then, the genome was searched for clusters containing at least two ASEGs within a 1 Mb region. A total of 35, 11, and 51 clusters of ASEGs were identified in BC/CB, MC/CM, and BM/MB crosses (Tables S1–S3), which is significantly higher than the numbers expected by chance (Fisher test; *p*-value < 0.001). For example, five genes (*Zm00001d006941*, *Zm00001d006942*, *Zm00001d006943*, *Zm00001d006944*, and *Zm00001d006945*) were located within ~17 kb on chromosome 2 from 219,959,370 to 219,976,357 bp. Interestingly, all five genes preferred to express the CAU5 allele in the BC/CB hybrid (Table S1).

### 2.2. Most of the ASEGs Were Consistent ASE across Different Tissues

**Figure 2:**
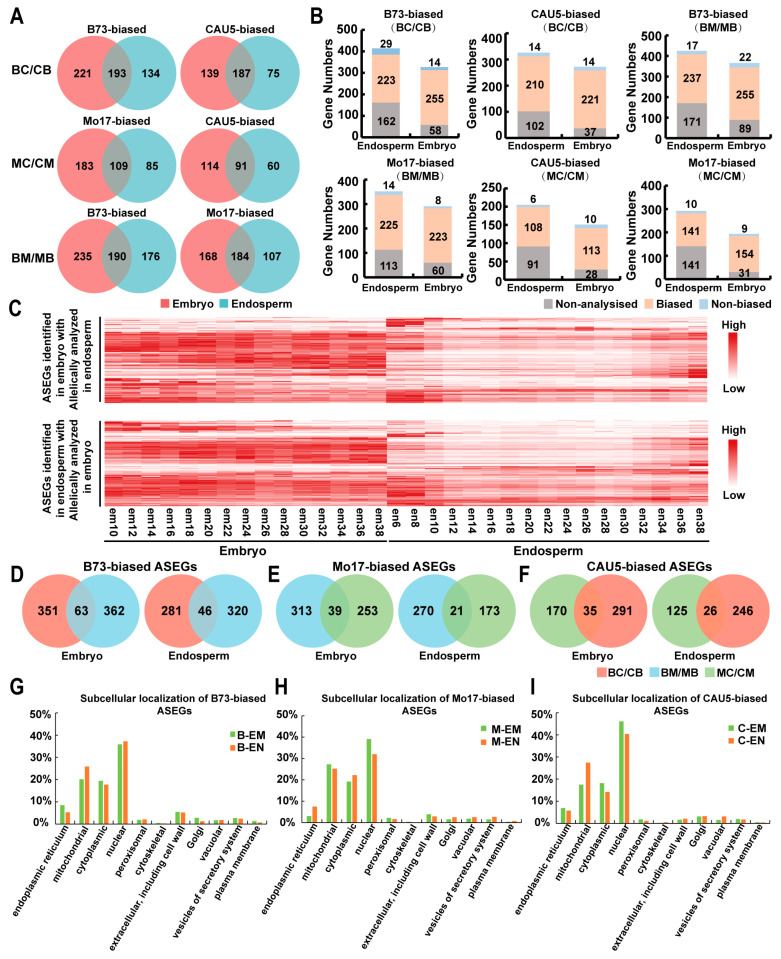
Comparison of ASEGs between tissues or hybrids. (**A**) Venn diagram analysis of the ASEGs identified in embryo and endosperm from a hybrid cross. (**B**) Comparison of ASEGs in two tissues from one hybrid cross. Non-biased: genes that do not show allelically biased expression (Chi-square (q > 0.05)). Non-analyzed: genes without sufficient read counts. Biased: genes showing significant allele-biased expression (Chi-square (2:1, q < 0.05)). (**C**) The expression of the ASEGs that can be allelically analyzed in both embryo and endosperm. (**D**–**F**) Overlap analysis of ASEGs identified in the same tissue from different hybrid crosses. (**G**–**I**) Subcellular localization of proteins encoded by ASEGs exhibited consistency in the same tissue from different hybrid crosses.

To increase the precision of the subsequent analysis, only ASEGs that reached a nine-fold difference between the reads of the two parents were used. First, we examined the genes that show consistent ASE across tissues in one hybrid cross. As visualized in the Venn diagram, approximately half of the ASE was consistent in both the embryo and endosperm of a hybrid cross (Figure 2A). For example, in BC/CB crosses, a comparison of ASEGs revealed 380 genes (51% in embryo and 63.4% in endosperm) that showed a consistent direction of expression bias in two tissues (embryo and endosperm), which included 193 genes that showed B73-biased expression and 187 genes that showed CAU5-biased expression (Figure 2A). Furthermore, ASEGs were found in a single tissue that usually did not have informative SNPs or had insufficient reads to identify whether they were ASEGs in other tissues (Figure 2B). For example, among the 414 B73-biased ASEGs identified in the BC/CB embryo, 223 genes (53.8%) exhibited B73-biased expression, only 29 genes (7.0%) were biallelically expressed, and 162 genes (39.1%) were not expressed or analyzed in BC/CB endosperm. Indeed, we found that the expression levels of ASEGs exhibited differences in the embryo and endosperm (Figure 2C and Figure S1). Therefore, for ASEGs analyzed and expressed in different tissues, most of them had consistent ASE across different tissues from one hybrid cross.

### 2.3. Half of the ASEGs Were Consistent ASE across Different Crosses

To further analyze whether some of the ASEGs showed consistency in different hybrid crosses, a Venn diagram of three hybrid crosses was generated. As illustrated in Figure 2D–F, only ~15% of ASEGs exhibited consistent ASE in the same tissue from all three hybrid crosses. Further analysis found that for ASEGs analyzed or expressed in different hybrid crosses, approximately half exhibited ASE from some genotypes but not others (Figure S2). For example, among the 414 B73-biased ASEGs identified in BC/CB embryos, 136 genes (32.8%) exhibited B73-biased expression, 99 genes (23.9%) were biallelically expressed, and 179 genes (43.2%) were not expressed or not analyzed in the BM/MB embryo.

The subcellular locations of ASEG encoded proteins that exhibited consistent ASE in the same tissue from the three hybrid crosses were then analyzed on the website of GenScript-PSORT II (https://www.genscript.com/psort.html?src=leftbar, accessed on 20 November 2022, Figure 2G–I). These ASEGs were separated into various subcellular locations, and nearly 40% of the ASEGs were located in the nucleus. For example, 414 B73-biased ASEGs identified in BC/CB embryos were mainly located in the nucleus (35.8%), followed by mitochondria (20.1%) and cytoplasm (19.4%), and the rest were distributed in several other organelles.

### 2.4. Mutant of One ASEG Zm00001d046765 Affects Kernel Development

To explore the function of these ASEGs in the development of embryos and endosperm of maize, we performed gene ontology (GO) analysis by distinguishing ASEGs in different genotypes and tissues (Figure S3). Three enriched GO terms for molecular functions, including the tricarboxylic acid cycle, aerobic respiration, and energy derivation by oxidation of organic compounds, were detected in B73-biased ASEGs identified in the embryos from BC/CB. A GO term for molecular functions, ADP binding, was detected in Mo17-biased ASEGs identified in embryos from BM/MB. CAU5-biased and Mo17-biased ASEGs in endosperm from MC/CM were enriched in the nuclear envelope and cytosol, respectively. Hence, GO analysis indicated that ASEGs have important roles in biosynthesis, development, and regulation. However, limited GO terms were common to ASEGs identified in different genotypes and tissues, suggesting that allele-specific genes have different roles in different genomic backgrounds.

**Figure 3:**
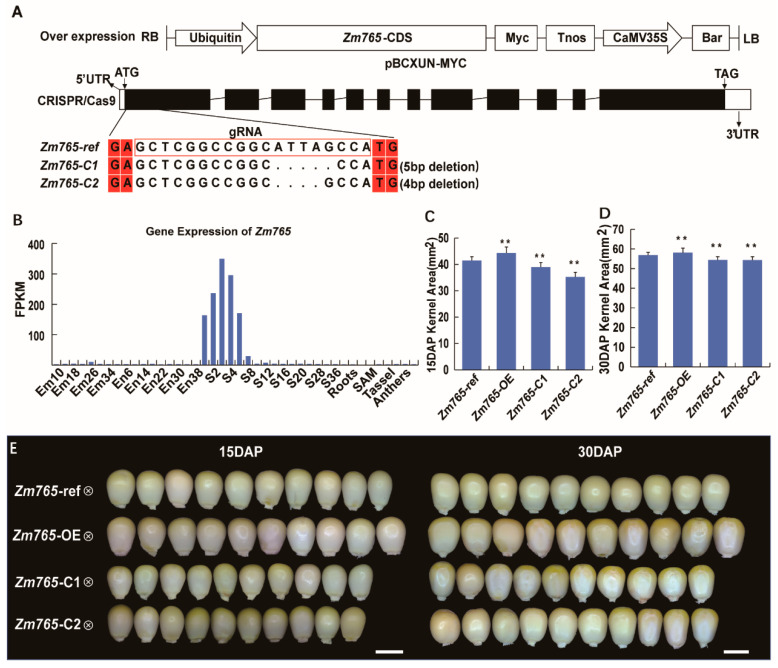
Phenotype analysis of *Zm765*. (**A**) Carrier structure of the *Zm765* overexpression and CRISPR/Cas9 lines. (**B**) Gene expression pattern of *Zm765*. (**C**) Comparison of the kernel area between the transgenic receptor line and two transgenic lines at 15 DAP. (**D**) Comparison of the kernel area between the transgenic receptor line and two transgenic lines at 30 DAP. (**E**) Kernel phenotypes of the transgenic receptor line and two transgenic lines at 15 DAP and 30 DAP. Left Bar = 4 mm, Right Bar = 5 mm, Significant differences were analyzed by two-tailed Student’s t tests (** *p* < 0.01).

Then, an ASEG, *Zm00001d046765* (*Zm765*), was selected for further phenotype analysis. *Zm765* is a B73-biased ASEG detected in the BC/CB embryo and is highly expressed in the early period of the kernel, which encodes a glycosyl hydrolase of unknown function. Therefore, we focused on comparing the kernel phenotypes of the overexpression lines and mutant lines with the transgenic receptor line to determine whether abnormal kernel phenotypes occurred during development. Both the area of the immature 15 DAP kernels and the mature 30 DAP kernels in the mutant lines showed a significant decrease compared to those in the transgenic receptor line (*p*-value < 0.01, Student’s test) (Figure 3). Then, we used transgene technology to create an overexpression line (*Zm765*-OE) to further analyze the function of *Zm765*. As shown in Figure 3C–E, at 15 and 30 days after self-pollination, the kernel areas of the *Zm765* overexpression lines were significantly larger than those of the transgenic receptor line at the corresponding period, which indicated that *Zm765* may participate in the development of the kernel.

### 2.5. The Allele Pattern of Methylation Level around ASEG

**Figure 4:**
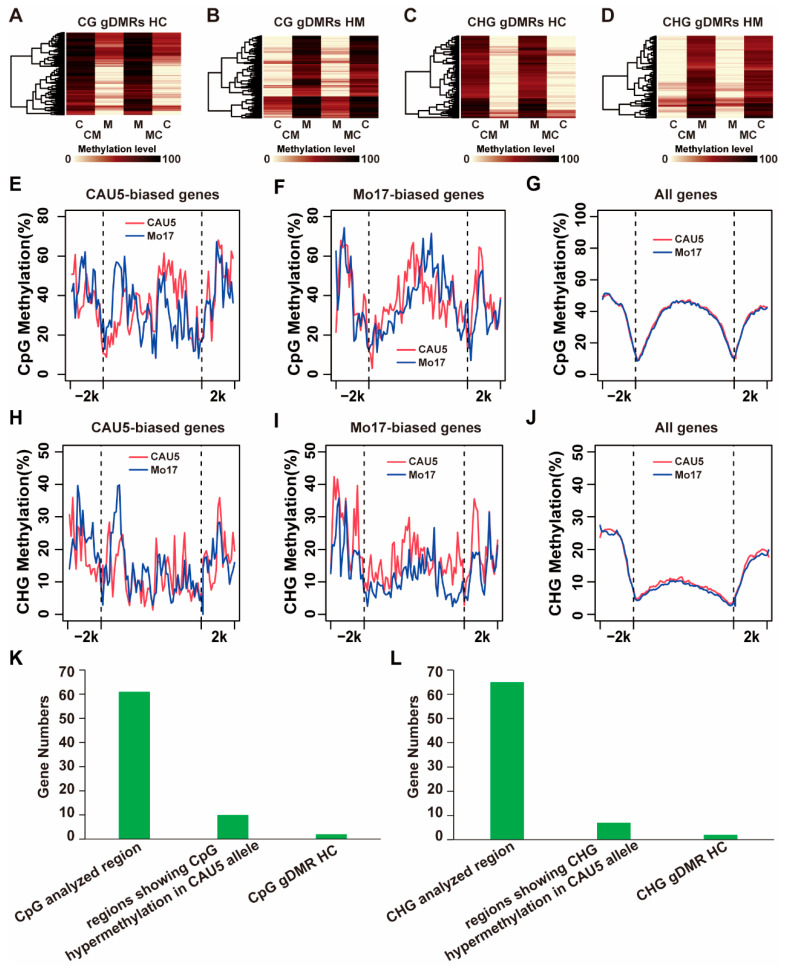
Allele DNA methylation pattern of ASEGs in endosperm of the MC/CM hybrid. (**A**–**D**) Heatmap of CG methylation levels between alleles of CAU5 and Mo17 reciprocal crosses at CG_pDMRs identified in MC/CM. (**E**–**G**) Differential CpG DNA methylation levels between two parental genomes for ASEGs identified in the endosperm of MC/CM. The red and blue lines represent the parent CAU5 and parent Mo17 methylation levels, respectively. The gene body regions were divided into 60 bins, and the upstream and downstream regions were divided into 20 bins. The average methylation levels were calculated with total C reads/total (C + T) reads in each bin. (**H**–**J**) Differential CHG DNA methylation levels between two parental genomes for ASEGs identified in MC/CM endosperm. (**K**) The overlap between ASEGs and CpG_gDMRs in MC/CM endosperm. (**L**) The overlap between ASEGs and CHG_gDMRs in MC/CM endosperm.

Based on the MethylC-seq performed for MC/CM endosperm, we scanned the genome to identify genotype-dependent differentially methylated regions (gDMRs) using a sliding window strategy in MC/CM endosperm (see Section 4). As a result, 1225 gDMRs were identified in the CG context (CG_gDMRs) (Table S4), including 649 CG_gDMRs showing hypermethylation in the CAU5 allele (CG_gDMRs_HC) and 576 CG_gDMRs showing hypermethylation in the Mo17 allele (CG_gDMRs_HM). In the CHG context, 307 CHG_gDMRs (gDMRs in the CHG context) were identified in MC/CM endosperm (Table S5), including 169 CHG_gDMRs hypermethylated in the CAU5 allele (CHG_gDMRs_HC) and 138 CHG_gDMRs hypermethylated in the Mo17 allele (CHG_gDMRs_HM). In Figure 4A–D, the allelic methylation pattern in the gDMRs region identified in MC/CM endosperm is shown.

The availability of ASEG and DNA methylome data in MC/CM allowed us to investigate the relationship between epigenetic modification and genotype-dependent allelic expression in maize. First, the patterns of allele DNA methylation at ASEGs were determined in the MC/CM endosperm (Figure 4E–J). As a result, in the context of CG, the methylation levels of the activated allele were slightly lower than those of the silenced alleles in the 5′ portion of the gene body regions (Figure 4E,F). However, the methylation levels of the activated allele were slightly higher than those of the silenced alleles in the 3′ portion of the gene body regions (Figure 4E,F). The levels of DNA methylation between two alleles of all genes were similar (Figure 4G). In the CHG context, similar results were observed. Then, we analyzed the association of gDMRs with ASEGs. Approximately 3% of ASEGs overlapped with CG_gDMRs or CHG_gDMRs (Figure 4K,L and Figure S4). For example, among 65 Mo17-biased ASEGs in the MC/CM endosperm that overlapped with the analyzed methylation region in the CHG context, 7 ASEGs overlapped with regions exhibiting hypermethylation in the CAU5 allele, and 2 ASEGs overlapped with CHG_gDMR showing hypermethylation in the CAU5 allele (Figure S4).

## 3. Discussion

### 3.1. ASEGs in the Different Tissues and Hybrid Crosses

To explore global ASEGs in hybrid maize and reveal the mechanism of differential expression in embryo and endosperm from F1 hybrids, three maize inbred lines (B73, Mo17, and CAU5) were chosen to generate three reciprocal crosses, BC/CB, MC/CM, and BM/MB. The proportion of genotype-dependent ASEGs identified in the three F1 hybrids did not differ significantly, which is similar to a previous report in rice and maize hybrids [16,41]. When ASEGs are compared in two tissues and three hybrids, ASEGs can be classified into two major patterns: consistent ASEGs and inconsistent ASEGs. In embryo and endosperm, most ASEGs were consistent in the same hybrid. Such a consistent biased expression of the genes would result in partially to fully dominant effects on the traits regulated by the genes [22]. However, in the same tissue from different hybrids, half of the ASEGs were inconsistent. Therefore, these results implied that the regulatory mechanism for the allele-specific expression of genotype-dependent ASEGs was mainly influenced by genetic variations [40,42]. In addition, ASEGs identified in three hybrid crosses also tend to be clustered in the genome. Moreover, the expressed directions of ASEGs located in one cluster were the same (Tables S1–S3), which indicated that the cause and regulatory mechanism of one ASEGs clusters might be the same. For an inbred line to cross with different inbred lines, the favorable allele of the genes can be variable, and the hybrid can make use of the favorable allele of the genes and express them at high levels.

### 3.2. The Potential Function of Genotype-Dependent ASEGs

The observed genotype-dependent ASEGs in the embryo and endosperm tissue of hybrid maize could represent a common mechanism of complementary allelic effects in hybrids and show the importance of the parental genotype in both cross-breeding and hybrid breeding [41,43]. The function of genotype-dependent ASEGs was involved in plant development and resistance to stress [44,45]. For example, *Ghd7* (a major QTL for grain number, plant height, and heading date) is present in rice-inbred MH63 but absent in inbred ZS97 and exerts a large pleiotropic dominance effect on all traits [22]. In our study, *Zm765*, expressed in B73 allele but silenced in CAU5 allele in BC/CB embryo, contributed to the development of maize kernel. In further work, whether *Zm765* exerts a pleiotropic dominance effect will be investigated. In addition, the GO annotation of genotype-dependent ASEGs was mainly enriched in metabolic pathways of substances and energy, such as the tricarboxylic acid cycle (TCA). TCA is the final oxidation pathway for glucose, fats, and amino acids and is the most important source of ATP production in cells [46]. Previous work also suggested that the TCA cycle can regulate plant reproductive development [47]. Therefore, our results indicated the importance of the parental genotype in the superior performance of the hybrid.

### 3.3. Methylation Plays an Important Role in the Regulation of Allelic Expression of Genotype-Dependent ASEGs

In recent work, ASE was negatively associated with allele-specific methylation (ASM) in CHG [38], indicating a specific pattern of DNA methylation reprogramming in hybrid rice and pointing to the role of parental CHG methylation divergence in ASE, which is associated with variation in phenotypes and hybrid vigor in several species of plants. In our study, although hypermethylation at CG and CHG repressed allele expression from one parent line, the relationship between ASEG and CHG_gDMR, but not CG_gDMR, was significantly higher than that of all genes. This is consistent with the finding that the silent maternal allele of paternally expressed genes in the endosperm of Arabidopsis lyrata is marked by hyper CHG methylation [48]. The present results indicate that CHG methylation of the allele-specific gene body is likely to be inherited from the parental epigenomes and is maintained or reinforced in an allele-specific manner in the hybrid and during development. Of course, only 10% of ASEGs overlapped with gDMRs. Except for DNA methylation, extensive allele-level histone modification was correlated with genome-wide changes in the allelic expression of genes [25,49]. Hence, the regulation for allele-specific expression of ASEGs was complex and should be explored with more datasets in the future.

## 4. Materials and Methods

### 4.1. Plant Materials

The hybrid lines B73(♀) × Mo17(♂), Mo17(♀) × B73(♂), B73(♀) × CAU5(♂), CAU5(♀) × B73(♂), CAU5(♀) × Mo17(♂), Mo17(♀) × CAU5(♂) were obtained from the inbred lines B73, Mo17, and CAU5 in the summer of 2021 at the experimental station of Shenyang Agriculture University in Shenyang, Liaoning. The ears and tassels of the three lines were bagged with kraft paper one day prior to pollination. The next day, each paper bag was patted to collect pollen from one parent, which was used to pollinate the ear of the other parent. After 11 days, the ears of six reciprocal crosses (BM, MB, BC, CB, MC, and CM) were collected. In this study, BM /MB represents the crosses of B73 × Mo17 and Mo17 × B73, BC /CB represents the crosses of B73 × CAU5 and CAU5 × B73, and MC /CM represents the crosses of Mo17 × CAU5 and CAU5 × Mo17.

### 4.2. Library Construction for RNA-Seq and MethylC-Seq

The embryo and endosperm samples were isolated using a Quick RNA Isolation Kit (Huayueyang Biotechnology of Beijing, China). mRNA library construction and sequencing were conducted following the Illumina manufacturer instructions. Total RNA was extracted as input material for the RNA sample preparations. The NEB Next^®^ Ultra TM RNA Library Prep Kit from Illumina^®^ (NEB, San Diego, CA, USA) was used to generate mRNA libraries. High-throughput mRNA sequencing was performed using the Illumina NovaSeq 6000 platform, and 150 bp paired-end reads were generated for each library. An average of 3 Gb data for each replicate was obtained and used for the following analyses, providing sufficient sequencing depth for the imprinting analysis.

Genomic DNA was extracted by the DNeasy plant mini kit (QIAGEN, Hilden, Germany). The purity was detected by a Nano Photometer^®^ spectrophotometer (IMPLEN, Westlake Village, CA, USA). Then, DNA fragments were treated with bisulfite (Accel-NGS Methyl-Seq DNA Library Kit for Illumina, Swift). Finally, the library quality of MethylC-seq was checked by the Agilent Bioanalyzer 2100 system. Pair-end sequencing was performed on the Illumina platform (Illumina, San Diego, CA, USA).

### 4.3. Read Mapping, Gene Expression Analysis, and SNP Calling

First, clean reads were mapped to the B73 reference genome (Version 4) using HISAT2 software with default parameters [50]. Cufflinks software (V2.2.1) was used to estimate the normalized gene expression values (FPKM) [51]. The calculated log2 (FPKM + 1) values were used to analyze the correlation coefficient between replicates. Hierarchical clustering analysis was performed on the relative expression value by setting the parameters’ average linkage and Euclidean distance using MeV (http://www.tm4.org/mev.html, accessed on 15 April 2022).

Resequencing reads of B73, Mo17, and CAU5 inbred lines were downloaded from NCBI (SRR12415217, SRR12415218, and SRR3124079). Reads were mapped using BWA with default parameters [52]. Samtools were used to exclude reads that were not uniquely mapped with the -q 20 parameter [52]. SNPs between B73, Mo17, and CAU5 inbred lines were called using Bcftools with default parameters [53].

### 4.4. Identification of Genotype-Dependent ASEGs

First, clean reads were mapped to the B73 reference genome (Version 4) using HISAT2 software (accessed on 12 March 2022) with default parameters [50]. To avoid bias, SNP sites were converted to CAU5 nucleotides to obtain the SNP-substituted CAU5 genome. All clean reads from three biological replicates of each sample were mapped to the B73 (Version 4) and SNP-substituted CAU5 genomes using HISAT2 with default parameters. Samtools was used to exclude reads that were not uniquely mapped with the -q 20 parameter [53]. Three replicates from each sample were merged for further identification of the imprinted genes. According to the SNP information, the reads aligned at the SNP site were split into maternal or paternal alleles using Samtools mpileup. The maternal and paternal read counts of each gene were summed. If the sum of the read counts of the annotated genes at all SNP sites was ≥20, the gene’s imprinting status could be analyzed. The maternal-to-paternal allele ratio of the genes analyzed was determined using the χ2 test to detect the deviation of the maternal: paternal ratio from the theoretically suggested 1:1 ratio in the embryo and the 2:1 ratio in the endosperm. Finally, read counts from one parental allele were used to identify ASEGs at least two-fold, five-fold, or nine-fold higher than read counts from another parental allele.

### 4.5. GO Term Enrichment and Functional Category Analysis

GO analysis of ASEG was performed using Agri GO v2.0 (accessed on 20 July 2022) [54]. Only GO terms are displayed among cell components, molecular functions, and biological processes with significant (*p*-value < 0.05) enrichment compared to all genes.

### 4.6. Pipeline for MethylC-Seq Analysis

MethylC-seq reads were generated using the same workflow as in previous work. First, low-quality reads were filtered using SolexaQA [55]. The remaining reads were mapped to the B73 genome using Bismark [56]. The bulk methylation of endosperm was calculated by the ratio of Cs to all Cs and Ts from all CG, CHG, or CHH sites. Then, SNPs were used to separate allele-specific MethylC sequence reads from the hybrid endosperm. Only sites with at least five reads were used in subsequent analyzes. The same criteria were used to identify CG_gDMR and CHG_gDMR as in previous work. First, a sliding-window approach with a 200-bp window and 20-bp step was adopted throughout the genome. Only windows containing more than five CG/CHG sites supported with at least five reads were kept as CG/CHG analyzed regions. Second, the statistical significance of the allelic methylation bias in each window was assessed by the *p*-value using Fisher’s exact test. The resulting *p* values were converted to Q values. Finally, the gDMRs were identified according to the following criteria: FDR < 0.01; the methylation level between two alleles differed by >30%; and the hypermethylated alleles had methylation levels > 40% in the context of CG. The candidate gDMRs were then further filtered using a smaller window size of 50 bp, and gDMRs within 200 bp were merged.

### 4.7. Genetic Transformation of Maize

We prepared overexpression constructs for the genetic transformation of one ASEG, *Zm00001d046765* (*Zm765*). Full-length CDS (without stop codon) of *Zm765* was amplified from *Zm765* cDNA and cloned into the binary vector pBCXUN-MYC to generate the pOE *Zm765*-MYC construct driven by the ubiquitin promoter (using the primers Zm765-CDS-F/R listed in Table S6). Transformations using the overexpression construct were introduced into the maize receptor line KN5585 via Agrobacterium-mediated transformation, and we verified the transgenic positive line with the primer of Bar-F/R (Table S6) [57]. For the CRISPR/Cas9 gene-editing construct, a 19-bp sequence from the first exon of *Zm765* was selected as a guide RNA (gRNA) and introduced into the pBUE411 vector as previously described [58]. For transformations using the CRISPR/Cas9 construct, two homozygous knockout lines of this gene with insertions or deletions at the target sites were identified from the independent positive transgenic lines (T0) by PCR amplification and sequencing analysis (using the primer of Zm765-CDS-F/R listed in Table S6). Independent positive transgenic lines were obtained and self-pollinated to generate homozygous progenies for kernel phenotype analysis.

### 4.8. The Method of Measuring Kernel Area

One-third of the kernels in the middle of the ear of the six crossed ears (BC, CB, MC, CM, BM, and MB) at 15 DAP and 30 DAP were separated and imaged under a light microscope (Olympus, Tokyo, Japan) one by one. Image J software was used to measure the area of each kernel.

### 4.9. Primers

All primers used in this study are listed in Table S6.

## 5. Conclusions

Allelic expression profiles in hybrid maize determined by RNA-sequencing technology demonstrated a type of genotype-dependent monoallelic expression gene in plants. The association analysis of DNA methylation and ASEGs indicated that epigenetic modifications have potential effects on the expression of ASEGs. In the future, we will pay more attention to the detailed functional analysis of the ASEGs detected in our study. Nonetheless, the ASEGs have provided an index of the genes for future studies, especially with respect to the genetic and molecular mechanism of heterosis, which would be helpful for hybrid breeding.

## Data Availability

Sequence data from this study can be found in the Sequence Read Archive at NCBI (SRA; http://www.ncbi.nlm.nih.gov/sra, accessed on 15 February 2022) under accession numbers and PRJNA765150.

## Supplementary Materials

The following supporting information can be downloaded at: https://www.mdpi.com/article/10.3390/ijms24054766/s1, Figure S1: The gene expression showing ASEGs that cannot be allelically analyzed in other tissues. Figure S2: Comparison of ASEGs in embryo and endosperm in three hybrids. Figure S3: The GO enriched terms of ASEGs. Figure S4: The overlap gene numbers between ASEGs and CpG_gDMRs in MC/CM endosperm. Table S1: List of ASEGs identified in BC/CB embryo and endosperm. Table S2: List of ASEGs identified in BM/MB embryo and endosperm. Table S3: List of ASEGs identified in MC/CM embryo and endosperm. Table S4: List of CpG_gDMRs identified in MC/CM endosperm. Table S5: List of CHG_gDMRs identified in the MC/CM endosperm. Table S6: Primers used in this experiment.

## Abbreviations

BM and MB	represent the crosses of B73 × Mo17 and Mo17 × B73, respectively.
BC and CB	represent the crosses of B73 × CAU5 and CAU5 × B73, respectively.
MC and CM	represent the crosses of Mo17 × CAU5 and CAU5 × Mo17, respectively.
DEGs	represent differentially expressed genes.
ASE	represents allele-specific expression.
ASEGs	represent allele-specific expression genes.
GO	represents gene ontology.
DAP	represents days after pollination.
WT	represents a wild type.
