# Supplementary material for: Characterization of Genes That Exhibit Genotype-Dependent Allele-Specific Expression and Its Implications for the Development of Maize Kernel
Source: Int J Mol Sci. 2023 Mar 1;24(5):4766. doi: 10.3390/ijms24054766 (PMC10002780; doi:10.3390/ijms24054766)
Supplement: Supplementary file 1 [file ijms-24-04766-s001.zip › Supplemental Figures.pdf]

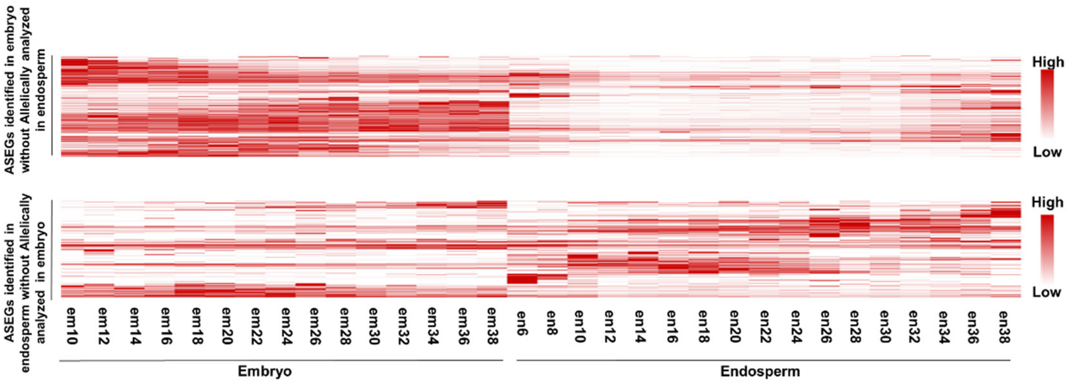

Figure S1: The gene expression showing ASEGs that cannot be allelically analyzed in other tissues.

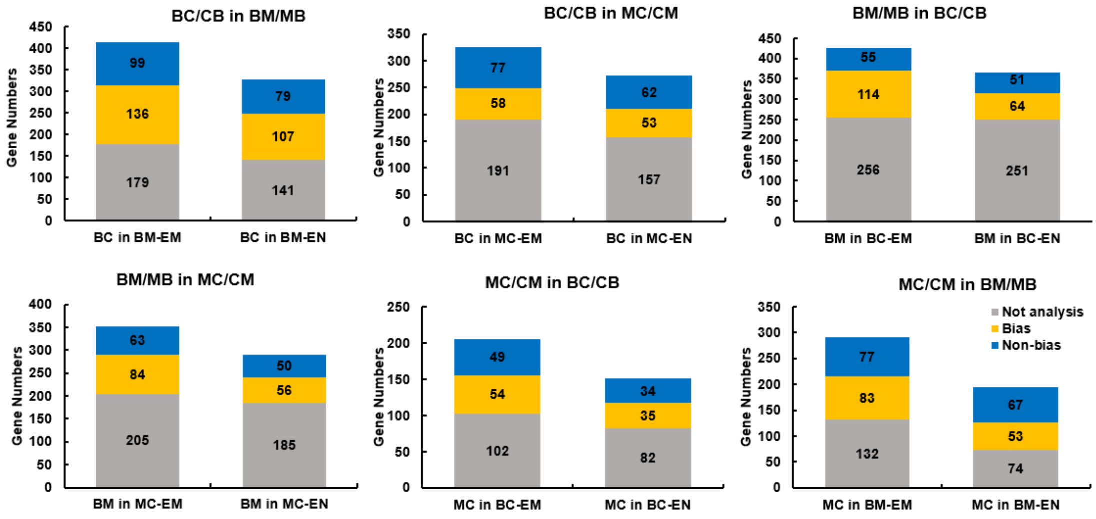

Figure S2: Comparison of ASEGs between embryo and endosperm in three hybrids. Non-biased: genes not showing allelically biased expression (Chi-square (q>0.05)). Non-analyzed: genes without sufficient read counts. Biased: genes showing significant allelically biased expression (Chi-square (2:1, q<0.05)).

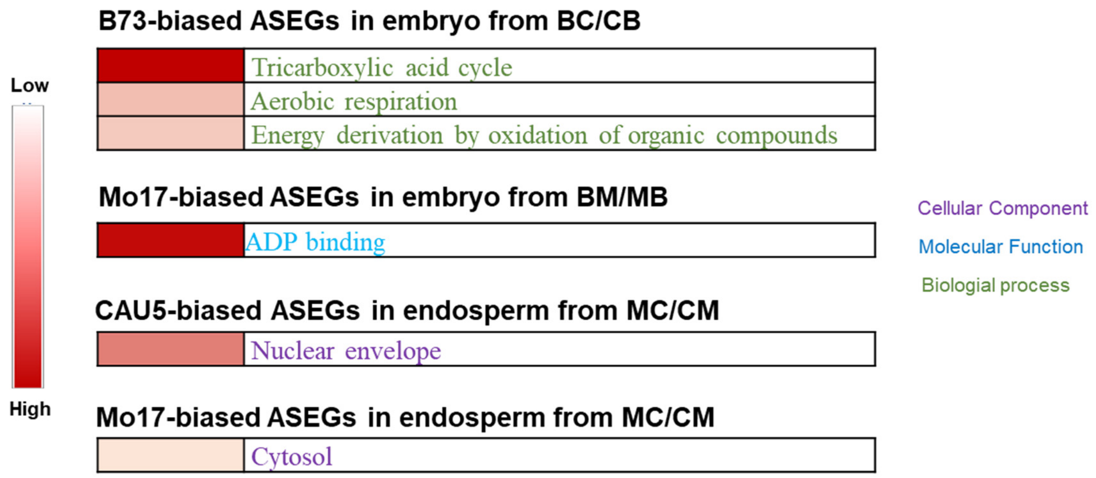

Figure S3: The GO enriched terms of ASEGs.

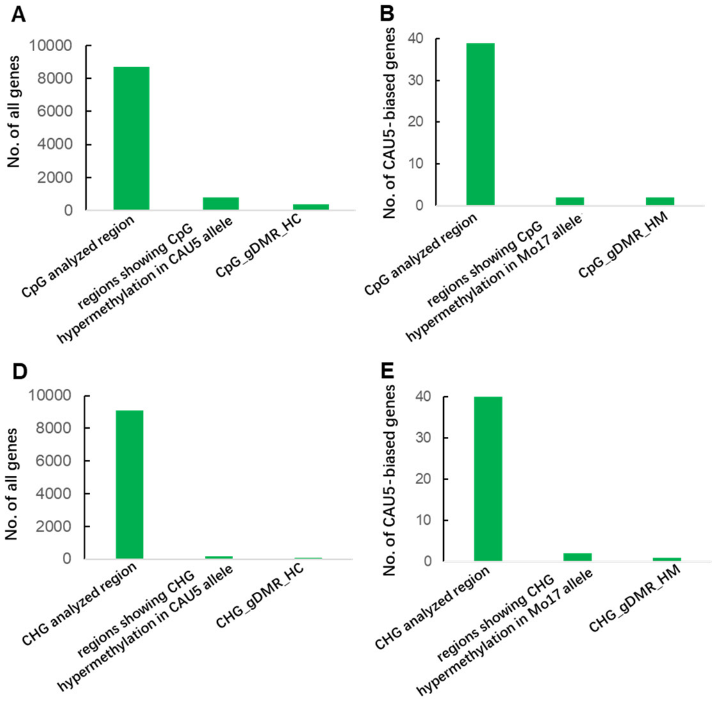

Figure S4: The overlap gene numbers between ASEGs and CpG_gDMRs in MC/CM endosperm.
